# Microbiome profiling of the onion thrips, *Thrips tabaci* Lindeman (Thysanoptera: Thripidae)

**DOI:** 10.1371/journal.pone.0223281

**Published:** 2019-09-30

**Authors:** Suresh J. Gawande, Sivalingam Anandhan, Ashish Ingle, Praveen Roylawar, Kiran Khandagale, Tushar Gawai, Alana Jacobson, Ramasamy Asokan, Major Singh

**Affiliations:** 1 ICAR-Directorate of Onion and Garlic Research, Rajgurunagar, Pune, India; 2 Department of Entomology and Plant Pathology, Auburn University, Auburn, Alabama, United States of America; 3 ICAR-Indian Institute of Horticultural Research, Hessarghatta Lake, Bengaluru, India; Oklahoma State University, UNITED STATES

## Abstract

The gut microbial community structure of adult *Thrips tabaci* collected from 10 different agro-climatically diverse locations of India was characterized by using the Illumina MiSeq platform to amplify the V3 region of the 16S rRNA gene of bacteria present in the sampled insects. Analyses were performed to study the bacterial communities associated with *Thrips tabaci* in India. The complete bacterial metagenome of *T*. *tabaci* was comprised of 1662 OTUs of which 62.25% belong to known and 37.7% of unidentified/unknown bacteria. These OTUs constituted 21 bacterial phyla of 276 identified genera. Phylum *Proteobacteria* was predominant, followed by *Actinobacteria*, *Firmicutes*, *Bacteroidetes* and *Cyanobacteria*. Additionally, the occurrence of the reproductive endosymbiont, *Wolbachia* was detected at two locations (0.56%) of the total known OTUs. There is high variation in diversity and species richness among the different locations. *Alpha*-diversity metrics indicated the higher gut bacterial diversity at Bangalore and lowest at Rahuri whereas higher bacterial species richness at *T*. *tabaci* samples from Imphal and lowest at Jhalawar. Beta diversity analyses comparing bacterial communities between the samples showed distinct differences in bacterial community composition of *T*. *tabaci* samples from different locations. This paper also constitutes the first record of detailed bacterial communities associated with *T*. *tabaci*. The location-wise variation in microbial metagenome profile of *T*. *tabaci* suggests that bacterial diversity might be governed by its population genetic structure, environment and habitat.

## Introduction

Bacterial communities in insects play an important role in their growth, development, immunological, physiological and morphological functioning. The majority of insects are believed to harbour heritable bacterial symbionts [1] that can be pathogenic, mutualist, or commensal, with some required for survival while others are not. Across Insecta, microorganisms have been reported to positively influence many functions, including the production of essential amino acids from nutrient poor diets [2], protection against toxic agents [3–5], aide in the production of honey [6], protection against parasitoids [7], virus transmission [8], insecticide resistance [9], degradation of phytotoxins and pesticides [10]. Conversely, some are reported to negatively impact insects by causing sterility and distorting sex ratios [11,12]. Despite their influence on important metabolic processes in the host, they have not been accurately profiled due to the difficulty in isolating and culturing many of the symbionts. Identification of symbionts has been improved with the availability of next generation sequencing technology, which, bypasses the need of isolating and culturing, can detect microbes present in very low amounts, and facilitates the study of the microbial community in its natural habitat with accurate taxonomic identification and their relative abundances [13]. Characterizing the diversity of symbionts is an important first step towards understanding their importance in the life history of organisms.

There is a growing area of interest regarding the presence and influence of heritable endosymbionts on the biology and ecology of economically important insects, and on the phenotypes that influence pest status. Several studies on endosymbionts have targeted pest species that damage crops by feeding on plants and transmitting plant pathogens such as aphids [14], whiteflies [15], and thrips [16]. The studies have identified endosymbionts that influence fitness [17], virus transmission [8], host plant preferences [18], protection from biological control agents [7], and insecticide resistance [9]. Endosymbionts are also of interest as targets for future pest control strategies that could be achieved by disturbing the essential symbionts of insect pests and symbionts which contribute to their pest status [19].

Onion thrips, *Thrips tabaci* Lindeman (Thysanoptera: Thripidae), is a globally important polyphagous insect pest. It has been collected from approximately 300 plant species that includes economically important crops such as leek, garlic, tobacco, cabbage, pea, melon, lettuce, potato, tomato, carnation, and cotton [20,21], and causes more than $1 billion losses worldwide to onion alone [22]. In addition to damaging crops through direct feeding, *T*. *tabaci* is a vector of the *Orthotospoviruses Iris yellow spot virus* (IYSV) and *Tomato spotted wilt virus* (TSWV) [23,24]. Onion thrips exhibits three different reproductive modes (thelytoky, arrhenotoky and deuterotoky) [25], host-associated lineages, variation in virus (TSWV) vector competence [26], heteroplasmy of *mtCOI* haplotypes [27] and develop resistance to insecticides quickly. Variation in these phenotypes may be due to selection on standing genetic variation but could be influenced by their microbiome. Insect bacterial communities may also influence their reproduction [28,29] and virus transmission efficiency of thrips [30].

To date, limited information is available regarding the endosymbiont profiles of this species; there is one study that examined one population using culturing methods [30] or only one bacteria species was examined^25^. The objectives of this study were to identify bacterial microbiota present in *T*. *tabaci* from ten distinct locations of India using the Illumina MiSeq platform to sequence the V3 region of 16s rRNA, and examine patterns of bacterial diversity within and among these locations.

## Materials and methods

### Ethics statement

*Thrips tabaci* has not been notified under any act or laws and rules thereof of the Government of India as an endangered or threatened species restricting or regulating its collection and observation. Therefore, permits were not required for collecting *T*. *tabaci* for the present study.

### Insect sampling

**Fig 1:**
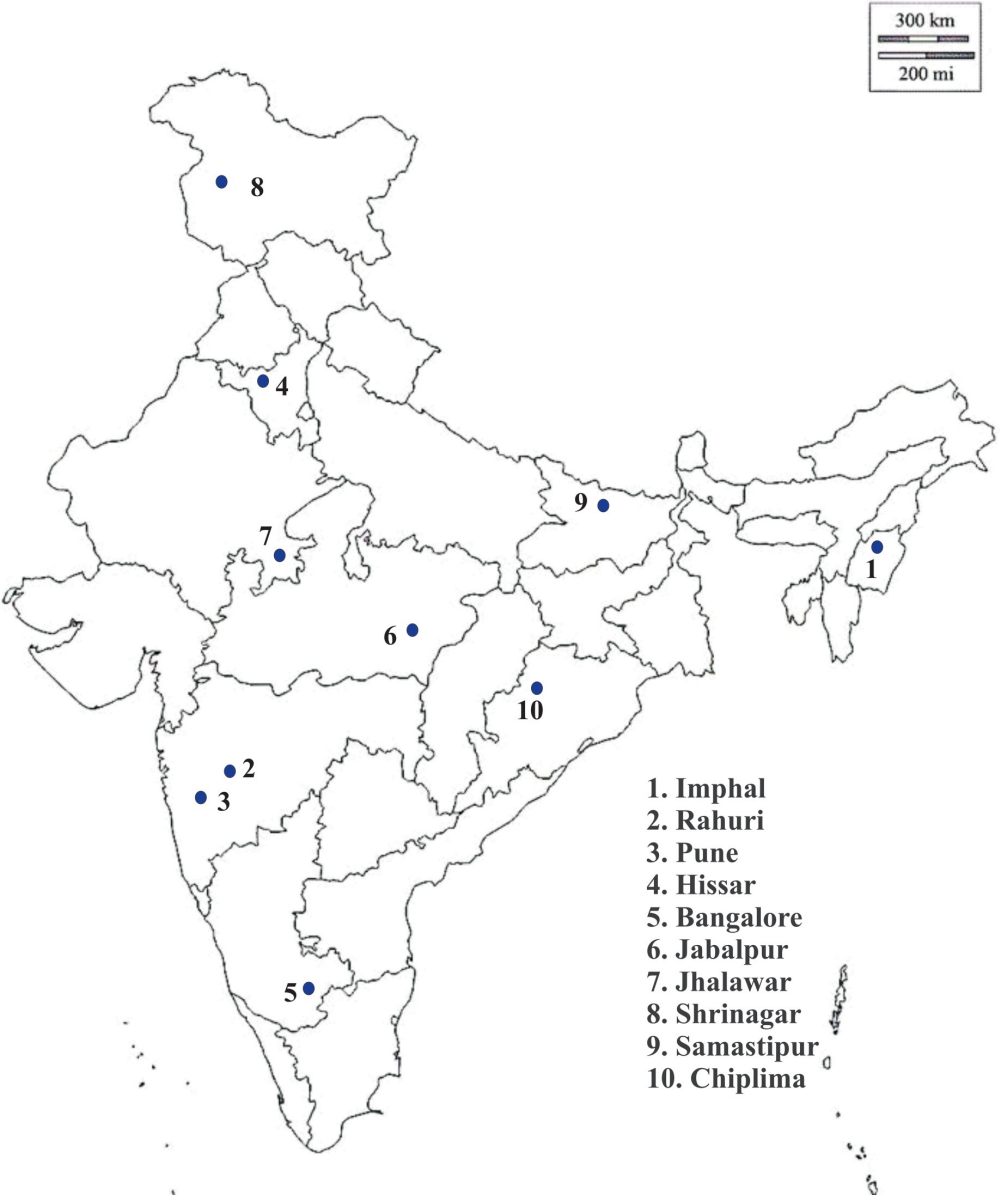
Locations, where *T*. *tabaci* were sampled, represent different climatic zones of India.

Adult *T*. *tabaci* were collected from 10 different states in India during *rabi* season (December-June) of the year 2013–2014. The states sampled encompassed different climatic zones in India, including temperate, tropical, and subtropical zones of the country (Fig 1). Approximately 500 thrips were collected from each state (50 thrips from each field, 10 fields per location), and were sampled within 1–2 days from each other. Adult thrips were dislodged from onion leaves with a fine brush into a 2 ml Eppendorf tubes containing 95% ethanol. To make certain offspring from different adults were collected, a distance of 1.5 m was kept between sampled plants. Voucher specimens are located at plant protection section, ICAR-Directorate of Onion and Garlic Research, Rajgurunagar, Pune.

### DNA extraction

Adult *T*. *tabaci* were surface sterilized by rinsing them with 100% ethanol. Genomic DNA was extracted from pool of 500 adult thrips from each location using a standard phenol-chloroform and ethanol precipitation method [31].

### Bacterial 16S rRNA gene amplification, library preparation and sequencing

16S rRNA gene sequencing was performed at SciGenom Labs Private Limited (Cochin, India) on an Illumina MiSeq 2 x 151 platform (Illumina, Inc., San Diego, CA, USA). Base quality score distributions, average base content per read and GC distribution in the reads were used as quality check parameters for sequences obtained from the sequencer (Illumina MiSeq).

### Bioinformatics and statistical analysis

To identify bacteria present in the samples sequence data needed to be filtered for quality, aligned into contigs, and blasted against existing bacterial sequence databases. An average of 361,241 paired end sequences with a length of 151bp were obtained from sequencing 10 samples (minimum 218,538; maximum: 499,241). Gut microbiome analysis was performed with QIIME2 2017.9 framework [32]. Raw sequences were quality filtered and denoised followed by chimera filtering with DADA2 [33] to obtain amplicon sequence variants (ASV). Low abundant ASVs with a total frequency less than ten were filtered out of the dataset. Taxonomy assignment was performed with a pre-trained Silva 132 99% OTUs based naïve Bayes classifier and confidence threshold of 0.7 using q2-feature-classifier plugin [34]. ASVs matching with chloroplast, mitochondria and eukaryotic sequences were removed from the database for downstream analysis. A taxonomic summary table was generated for each level of taxonomy for phylotype abundance.

Sequence alignment using MAFFT [35] was performed for all ASVs and used for the construction of phylogeny with fasttree2 [36]. Alpha rarefaction plotting was performed with a minimum and a maximum depth of 100 and 10000 respectively to identify an ideal sampling depth for further analysis. Three alpha diversity indices namely, Shannon, chao1, and simpson_e at ideal sampling depth have been estimated. The similarity of bacterial communities between samples (beta diversity) was quantified using a metric based on phylotype abundances. The distance matrix was generated using weighted UniFrac approach [37], and a jackknife test with 100 iterations was performed to construct a consensus UPGMA (Unweighted Pair Group Method with Arithmetic Mean) tree. Principal Coordinates Analysis (PCoA) was performed to generate a 3D PCoA plot in EMPeror [38].

ANCOM compares the log ratio of the abundance of each taxon to the abundance of all the remaining taxa one at a time [39]. ANCOM (ANalysis of Composition Of Microbiomes) was performed to identify taxa with differential abundance among sample groups. ANCOM is now incorporated into the QIIME suite for metagenome analyses. For ANCOM analysis samples were grouped into four climatic zones according to Köppen classification viz; (Monatne (MON): Shrinagar; Humid subtropical (HST): Hisar, Imphal, Jabalpur, Samastipur; Tropical wet and dry (TWD): Pune, Rahuri, Chiplima and Semi-arid (SA): Bangalore and Jhalawar.

## Results

### Sequencing data

The Illumina MiSeq sequencing of the V3 region of 16S rRNA gene of onion thrips from ten different locations yielded 218,538–499,241 raw reads per location. Nearly 80% of the total reads had Phred scores greater than 30 (>Q30; error-probability > = 0.001) indicating good quality of data. GC content of the reads ranged from 40 to 60% and after filtering contig length (~150bp). After quality filtering 39,976–140,603 reads per sample remained. Pre-processed reads from all samples were pooled for a total of 962,166 reads, and from them, a total of 1662 OTUs were identified (Table 1).

**Table 1:** Sequencing analysis of V3 region of 16S rRNA gene of *T*. *tabaci*.

Sample	Latitude/Longitude	No. of Reads	OTUs
Bangalore	13.135/77.496	112995	317
Chiplima	21.345/83.91	70134	122
Hisar	29.1491/75.7216	89733	185
Imphal	24.8170/93.9368	140603	234
Jabalpur	23.2072/79.9539	102781	221
Jhalawar	24.5399/76.1430	39976	39
Pune	18.8430/73.8848	139747	146
Rahuri	19.3490/74.6460	73581	45
Samastipur	25.9844/85.6744	101007	225
Srinagar	33.9842/74.7990	91609	128

### Microbiome profile of *T*. *tabaci*

**Fig 2:**
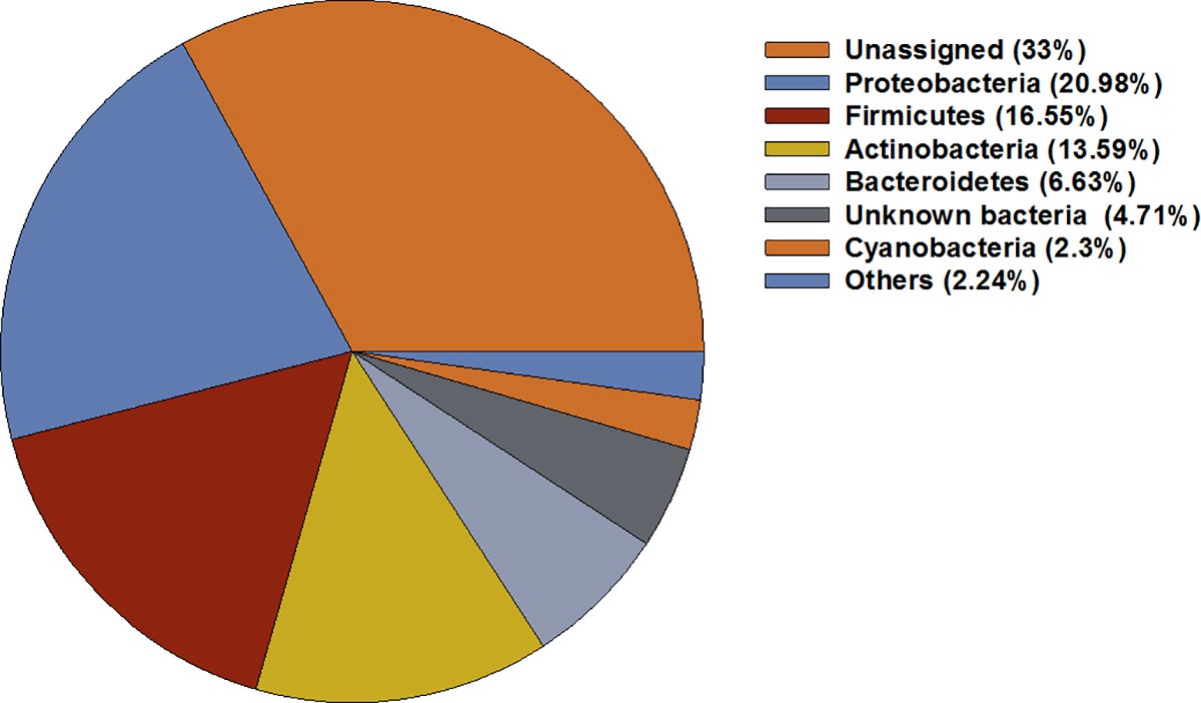
The relative abundance of dominant bacterial Phyla represented in *T*. *tabaci* samples collected from all ten locations across India.

**Fig 3:**
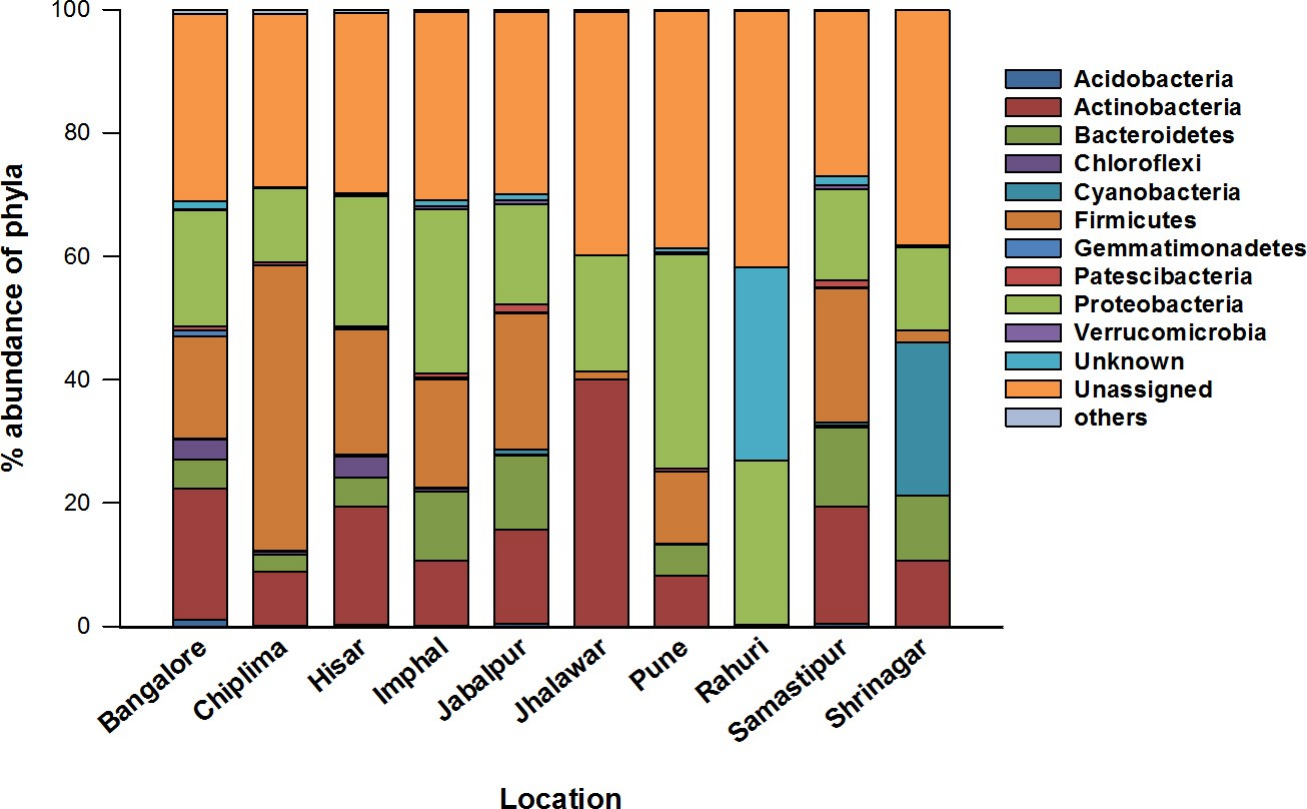
The abundance of different phyla of bacterial community of *T*. *tabaci* from ten different locations of India.

A total of 21 phyla, which constitute 62.25% of the total 16S rRNA gene data set, were detected from *T*. *tabaci* in this study (S1 File). The majority of bacterial contigs were of unknown origin (33%) while 4.7% were unknown bacteria. The contigs that do not have any alignment against the taxonomic database were categorized as “Unassigned”. From the identifiable bacterial sequences, the majority belonged to phylum *Proteobacteria*, followed by *Firmicutes*, *Actinobacteria*, *Bacteroidetes* and *Cyanobacteria* (Fig 2, Fig 3). Across all locations, these five phyla comprised more than 60% of the total microbiome and more than 96% of identified microbiome of *T*. *tabaci*.

At the bacterial class level, the *Proteobacteria* were comprised of 26 *Gamma*-, 7.1% *Alpha-proteobacteria*. In *Firmicutes*, classes *Bacilli* and *Clostridia* represented 14.1% and 10%, respectively. In the phylum *Actinobacteria*, classes *Actinobacteria* and *Coriobacteria* represented 16.8% and 3.5%, respectively. Class *Bacteroidia of* phylum *Bacteroidetes* constituted 10.6% of total identified microbiome and classes *Oxyphotobacteria* and *Melainabacteria* of phylum *Cyanobacteria* represented 3.4%, and 0.2%, respectively. At the genus level, a total 276 genera were identified from the present study. The highest number of genera are from phylum *Proteobacteria* (69) followed by *Firmicutes* (61), *Actinobacteria* (44), *Bacteroidetes* (29) and *Chloroflexi* (13). Among them, the genus *Streptococcus* (phylum *Firmicutes*) was most prevalent and comprised 8.03% of contigs, followed by *Pseudomonas* (5.4%) (phylum *Proteobacteria*), *Rosenbergiella* (4.5%) (phylum *Proteobacteria*), *Alistipes* (2.7%) (phylum *Bacteroidetes*) and *Saccharopolyspora* (2.05%) (phylum *Actinobacteria*). In addition, reproductive endosymbiont *Wolbachia* was recorded at 0.56% of the total OTUs. A few plant endophytes were also identified from gut of onion thrips in the present study such as *Actinomyces*, *Microbacterium*, and *Burkholderia* at very low levels.

### Microbiome diversity of *T*. *tabaci* at different location

**Fig 4:**
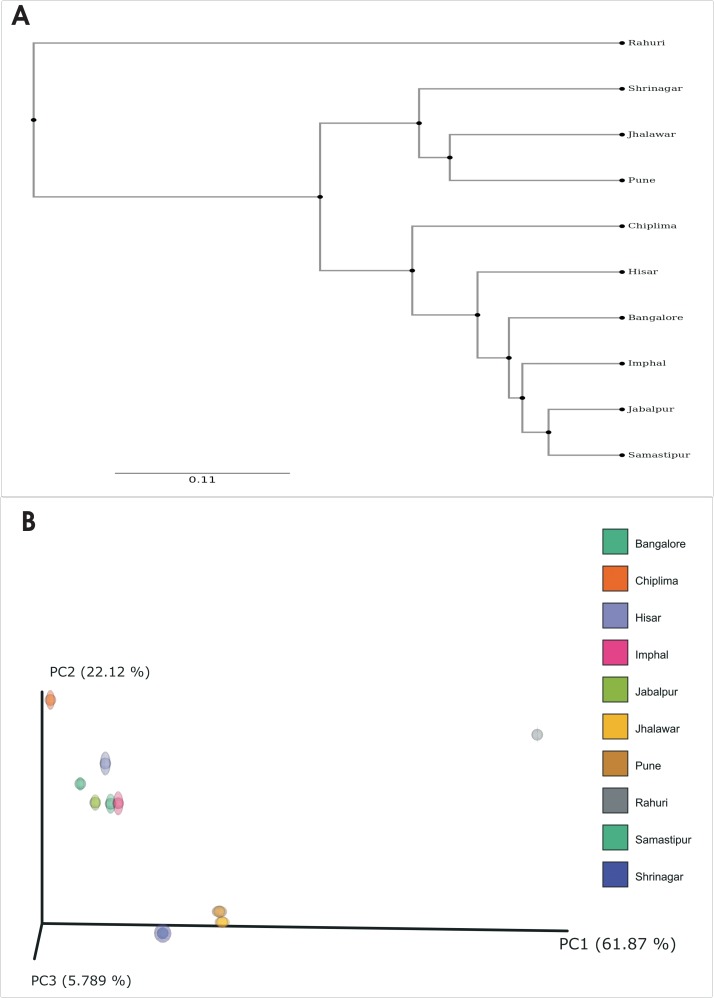
UPGMA tree (A) and PCoA plot (B) showing relationships between the gut bacterial communities from *T*. *tabaci* collected from 10 different geographic locations and based on β-diversity metrics calculated using UniFrac.

Alpha-diversity indices (Shannon, Simpson-e) describe the diversity of the microbial community at each sampling location and showed that bacterial diversity is higher in Bangalore, followed by Jabalpur and that the lowest bacterial diversity is observed in Rahuri. Another alpha diversity index (Chao1) describes the species richness at each sampling location. Among ten locations, bacterial richness is found to be highest in Bangalore and lowest in the Jhalawar (Table 2). Beta diversity considers the variations in bacterial community composition for different environments. Bacterial diversity among locations was assessed using both the weighted and unweighted UniFrac approach. UniFrac distances are based on the fraction of branch length of the 16S rRNA phylogenetic tree shared between two communities. The UPGMA tree and PCoA plots were constructed using the weighted UniFrac distance matrix. Locations were clustered based on the bacterial community structure. From PCoA plot it has been seen that there is high diversity among the bacterial communities of *T*. *tabaci* from different geographical location (Fig 4). PC-1 and PC-2 explain 61.87% and 22.1% cumulative variance in the microbiome of the *T*. *tabaci*, respectively. ANCOM analyses reveals two genera in Montane zone *Chryseobacterium* and *Exiguobacterium* found to be higher in Shrinagar location (S2 File, S3 File).

**Table 2:** Diversity indices calculated for microbial communities associated with *T*. *tabaci* from ten different locations of India.

Sample/Locations	Shannon	Chao1	Simpson_e
Bangalore	7.82	319.49	0.59
Chiplima	5.01	123.39	0.08
Hisar	6.49	189.44	0.20
Imphal	6.82	237.65	0.21
Jabalpur	7.17	227.70	0.49
Jhalawar	4.00	39.00	0.28
Pune	5.74	146.45	0.13
Rahuri	2.88	46.10	0.11
Samastipur	7.00	234.14	0.33
Shrinagar	5.56	131.25	0.14

## Discussion

Microorganisms exhibit a variety of interactions with their hosts and most of the time these interactions are beneficial to the insect [40]. Despite being an economically important pest, the information on insect-microbe interactions of *T*. *tabaci* is very limited. Recently, NGS has become the method of choice for insect microbiome analyses due to the ability to detect both culturable and non-culturable bacteria. The present study constitutes the first detailed examination of bacterial communities in *T*. *tabaci* using sequencing methods. In this study phyla *Proteobacteria*, *Actinobacteria*, *Firmicutes* and *Bacteroidetes* together constituted more than 90% of the total *T*. *tabaci* identified microbiome. These phyla are also reported to be the predominant in the microbiomes of other thrips species [16,41,42], several insects [43,44,45], and amphibians [46].

Phylum *Proteobacteria* was predominant at four of the ten reported locations. *Proteobacteria* plays an important role in carbohydrate degradation in Wood Borer, *Saperda vestita* [47], vitamins synthesis [48] and detoxification of pesticides fruit fly *Bactrocera dorsalis* [49,10]. The phylum *Actinobacteria*, was abundant at two locations and is reported to increase metabolic versatility and the ability to exploit the wide range of nutritional resources e.g. polysaccharides like cellulose [50] and hemicelluloses in termites [51] and production of secondary metabolites with antibiotic properties [52]. The third most prevalent phylum was *Firmicutes*, which predominated at three locations. Bacteria in *Firmicutes* were also abundant in guts of termites and honeybees [53,54]. Several studies in insects and animals have shown that *Firmicutes* increased the ability to metabolize food resources to increase energy conversion from diet [55], and assist in the digestion of cellulose and hemicelluloses [56]. *Bacteroidetes* were the fourth predominant phylum in present16S rDNA data set. The members of this phyla are known for their role in the production of enzymes such as glucanase, mannanase and xylanase that aid in complex carbohydrate metabolism [57,58]. Phylum *Cyanobacteria* was prevalent in one location, and these bacteria are associated with high levels of protein, vitamins, and microelements. They are known to release toxins during their life that lead to concentration dependent, and species-specific negative effects on animal feeders [59]. These five phyla were also found to be predominant in the microbiome of the other thrips species *Scirtothrips dorsalis* and *Hoplothrips* carpathicus [16,42].

We detected the genus *Wolbachia*, in two out of ten locations. *Wolbachia* infects over 40% of all arthropods [60] and is known to manipulate sex determination mechanisms and sex ratio by cytoplasmic factors, cytoplasmic incompatibility [61,62]. This is the first report of *Wolbachia* in *T*. *tabaci*. Studies have looked presence of *Wolbachia* in different thrips species but did not find it in *T*. *tabaci* [25,29]. *Wolbachia* has been reported in other thrips species such as *Echinothrips americanus*, *Gynaikothrips ficorum*, *Suocerathrips linguis* [29], *Thrips palmi* [63] and *Hoplothrips carpathicus* [42] but their role in *T*. *tabaci* is unclear.

Few plant origin endophytes such as *Actinomyces*, *Microbacterium*, and *Burkholderia* were recorded in the microbiome of *T*. *tabaci*. Theses endophytes might have been acquired by the insect during feeding on plants. Their occurrence in insects gut has been well documented in previous studies [64,65].

*Streptococcus*, *Saccharopolyspora*, *Phormidium*, *Pseudomonas*, *Prevotella*, *Serratia*, *Erwinia* and *Propionibacterium* were the most predominant genera in the microbiome of *T*. *tabaci*, and were also reported to be predominant in the microbiome of several other thrips species [16,66,67,30,68,42].

This study marks the first attempt to document the endosymbiont diversity associated with *T*. *tabaci* in India. Diversity indices showed that *T*. *tabaci* from Bangalore has the highest bacterial diversity and Imphal has the highest species richness. Further UPGMA and PCoA analyses showed bacterial communities structured by location. This spatial variation in the microbiome *T*. *tabaci* might be due to geographical location, climatic conditions and host phylogeny. In several insects, variation in the microbiome with respect to climatic conditions, the geography of habitat, and phylogeny of the insect have been documented [69,42,14].

## Conclusion

This paper described the microbiome of *T*. *tabaci* collected from the different geographical locations of India using NGS approach. Findings of the present study increased understanding of microbiome of *T*. *tabaci* as well as its variation with respect to geography and climatic conditions. Though it is first report of its kind in *T*. *tabaci*, for more in depth study of *T*. *tabaci* microbiome needs to be done at different developmental stages for better understanding of its role in development and growth of *T*. *tabaci*.

## Supporting information

S1 File Microbiome profile of *T*. *tabaci*. (XLSX)

S2 File Volcano plot of ANCOM analysis. (PNG)

S3 File Percentile abundances of taxa across the groups. (TSV)
