# Supplementary figures and images for: Microbiome profiling of the onion thrips, Thrips tabaci Lindeman (Thysanoptera: Thripidae)
Source: PLoS One. 2019 Sep 30;14(9):e0223281. doi: 10.1371/journal.pone.0223281 (PMC6768462; doi:10.1371/journal.pone.0223281)

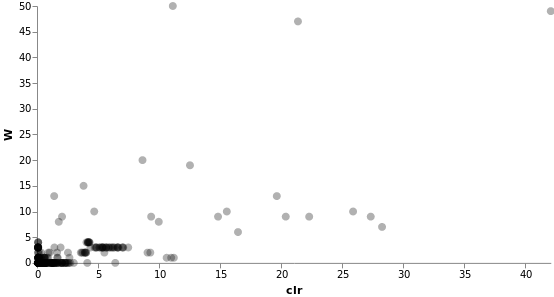

Supplement: S2 File — (PNG) [file pone.0223281.s002.png]
